# Assessment of the Ecological Association between Tobacco Smoking Exposure and Bladder Cancer Incidence over the Past Half-Century in the United States

**DOI:** 10.3390/curroncol30020154

**Published:** 2023-02-06

**Authors:** Thomas Seisen, Muhieddine Labban, Stuart R. Lipsitz, Mark A. Preston, Matthew Mossanen, Joaquim Bellmunt, Morgan Rouprêt, Toni K. Choueiri, Adam S. Kibel, Maxine Sun, Quoc-Dien Trinh

**Affiliations:** 1 Division of Urological Surgery, Brigham and Women’s Hospital, Harvard Medical School, Boston, MA 02115, USA; 2 Center for Surgery and Public Health, Brigham and Women’s Hospital, Harvard Medical School, Boston, MA 02215, USA; 3 Department of Urology, Pitié-Salpêtrière Hospital, APHP, Sorbonne University, GRC *n*°5 Predictive Onco-Urology, 75013 Paris, France; 4 Lank Center for Genitourinary Oncology, Dana-Farber/Brigham and Women’s Cancer Center, Harvard Medical School, Boston, MA 02115, USA

**Keywords:** epidemiology, incidence, lung neoplasms, prevalence, smoking, tobacco, urinary bladder neoplasms

## Abstract

Background: Since tobacco smoking represents the most established risk factor for bladder cancer, we sought to assess the ecological association between tobacco smoking prevalence and bladder cancer incidence and to contrast it with lung cancer. Methods: The annual overall tobacco smoking prevalence rates were extracted from the Report of the Surgeon General and the Center for Disease Control between 1953 and 1983. The overall age-adjusted incidence rates for bladder and lung cancers were derived from the Surveillance, Epidemiology, and End Results database between 1983 and 2013 (30-year latency period). Weighted least square regression models were used to assess bladder and lung cancer incidence rate differences (IRD) related to trends in tobacco smoking prevalence. A Wald test was used to compare whether the prevalence of tobacco smoking, as an explanatory variable, differentially predicts bladder versus lung cancer incidence rates. Results: The associations between tobacco smoking prevalence and bladder cancer incidence were not significant in the overall (IRD = +0.04; 95%CI (−0.14; +0.22); *p* = 0.63), male (IRD = +0.07; 95%CI (−0.09; +0.23); *p* = 0.37), or female (IRD = +0.12; 95%CI (−0.01; +0.25); *p* = 0.06) populations. There was an association between tobacco smoking prevalence and lung cancer incidence in the overall (IRD: +3.55; 95%CI ( +3.09; +4.00); *p* < 0.001), male (IRD: +4.82; 95%CI (+4.44; +5.20); *p* < 0.001), and female (IRD: +3.55; 95%CI (+3.12; +3.99); *p* < 0.001) populations. The difference between the observed associations of tobacco smoking prevalence with bladder versus lung cancer incidence was also significant in all examined populations (*p* < 0.001). Conclusions: Variations in tobacco smoking prevalence only partially explained the trends in the incidence of bladder cancer, indicating that its etiology is complex.

## 1. Introduction

Bladder cancer is a lethal disease; it was estimated that there would be 81,180 new cases and 17,100 new deaths in 2022 [1]. Over the last thirty years, no improvements in bladder cancer-specific survival have been observed [2]. According to the American Cancer Society, smokers are at least three times more likely to develop bladder cancer compared to non-smokers [3]. To date, bladder cancer remains a leading cause of cancer-related mortality and one of the most expensive malignancies to treat in the United States [4].

Tobacco smoking has been purported to be the most important risk factor for bladder cancer [5], as case–control studies have historically suggested a near-causal relationship between smoking and bladder cancer [6]. However, this causal association appears to be more complex than previously thought. There is evidence that exposure to aromatic amines through smoking may cause DNA adduction and mutagenesis [7,8,9]. In recent meta-analyses, the risk of developing bladder cancer was found to be higher among current smokers compared to former smokers [10,11]. Moreover, reports showed that, for an equal total exposure in pack-years smoked, smoking less for a longer duration is more harmful than smoking more for a shorter duration [11]. Furthermore, while the risk of developing bladder cancer among former smokers decreases with the number of years since quitting smoking [12], it remains higher compared to never-smokers; this is true even among former smokers who quit smoking over 15 years ago, suggesting an early-stage irreversible effect of tobacco smoking [13].

The harmful effects of tobacco smoking prompted the United States government to initiate large interventional programs and enforce stringent laws against tobacco smoking [14,15]. These initiatives resulted in a significant drop in tobacco smoking after peaking in the 1950s among men and in the 1960s among women [16,17]. Therefore, the trends in the prevalence of tobacco smoking over the past half-century provide a unique opportunity to investigate the ecological association between tobacco smoking and bladder cancer incidence.

In this context, our primary objectives were to (1) examine the association between the prevalence of tobacco smoking and age-adjusted incidence rates for bladder cancer in the United States, and (2) compare trends in the age-adjusted incidences rates for bladder cancer with those for lung cancer, given its unquestioned near-causal relationship with smoking. Assuming that tobacco smoking is the most important risk factor for bladder and lung cancer, our hypothesis is that trends in the age-adjusted incidence rates of both malignancies are closely associated to trends in the prevalence of tobacco smoking.

## 2. Materials and Methods

### 2.1. Tobacco Smoking Prevalence

Our primary independent variable was the annual prevalence of tobacco smoking between 1953 and 1983. A current smoker was defined as an individual ≥ 18 years old who reported smoking ≥ 100 cigarettes during their lifetime, and who, at the time they participated in the National Health Interview Survey, reported smoking every day or some days [18]. Since there is no single data source covering 1953 to 1983, we relied on both the Reports of the Surgeon General [19] and the Center for Disease Control [20] to derive the overall and gender-specific annual prevalence of tobacco smoking in the United States over this period.

### 2.2. Age-Adjusted Bladder and Lung Cancer Incidence Rates

Our primary dependent variable was the bladder cancer annual age-adjusted incidence rates (per 1000 person-years) between 1983 and 2013. We used the Surveillance, Epidemiology, and End Results (SEER)–9 registries, which provide a population-based sample representing around 28% of the United States population [21], to obtain age-adjusted incidence rates for bladder cancer by identifying adults ≥ 40 years old with a histologically confirmed diagnosis of primary bladder cancer (ICD-O-3 site codes: C67.0–C67.9). As a comparator, we obtained the age-adjusted incidence rates for primary lung cancer between 1983 and 2013 by identifying adults aged ≥ 40 years old with a histologically confirmed diagnosis of lung cancer (ICD-O-3 site codes: C34.0–C34.9). Since SEER is a population-based dataset with cancer-specific outcomes, it is a reliable resource for epidemiological studies [21].

### 2.3. Statistical Analyses

The annual prevalence of tobacco smoking (1953–1983), as well as the annual age-adjusted incidence rates for bladder and lung cancers (1983–2013), were graphically depicted for the overall population and then stratified according to gender. A 30-year latency period between the prevalence of tobacco smoking and cancer incidence was chosen based on the ‘tobacco epidemic’ theory, which states that smoking-related deaths occur between 30 and 40 years after initial smoking exposure [22]. Linear regression models were fitted to generate the annual percent changes (APCs) of tobacco smoking prevalence per year between 1953 and 1983. The APCs for bladder and lung cancer incidence rates, as well as the corresponding 95% confidence intervals (CIs), were obtained from SEER*Stat software.

To test the associations between the prevalence of tobacco smoking and incidence rates for bladder and lung cancer, we modeled the incidence rates of each malignancy (per 100,000 person-years) separately as a linear regression function of tobacco smoking prevalence (in percent)—first within the overall population and then within gender-specific populations. Therefore, the regression coefficients corresponded to the incidence rate differences (IRDs), which can be interpreted as the increasing or decreasing number of malignant cases per 100,000 person-years for every 1%-point increase in tobacco smoking prevalence. These regression coefficients were estimated using weighted least squares, with weights equal to the inverse of the estimated variance of the yearly incidence rate for each malignancy (estimated variance obtained from SEER*Stat software).

A Wald test was used to compare whether the prevalence of tobacco smoking, as an explanatory variable, differentially predicts bladder versus lung cancer incidence rates. Finally, the proportion of smoking-related changes in the incidence of each malignancy was estimated using the R-squared statistic derived from the weighted least squares regression model. All statistical analyses were performed using Stata^®^ (Version 14.0, StataCorp LP, College Station, TX, USA). Two-sided statistical significance was defined as a *p* value < 0.05.

## 3. Results

### 3.1. Overall Population

#### 3.1.1. Annual Percent Changes in Tobacco Smoking Prevalence and Cancer Age-Adjusted Incidence Rates

**Figure 1:**
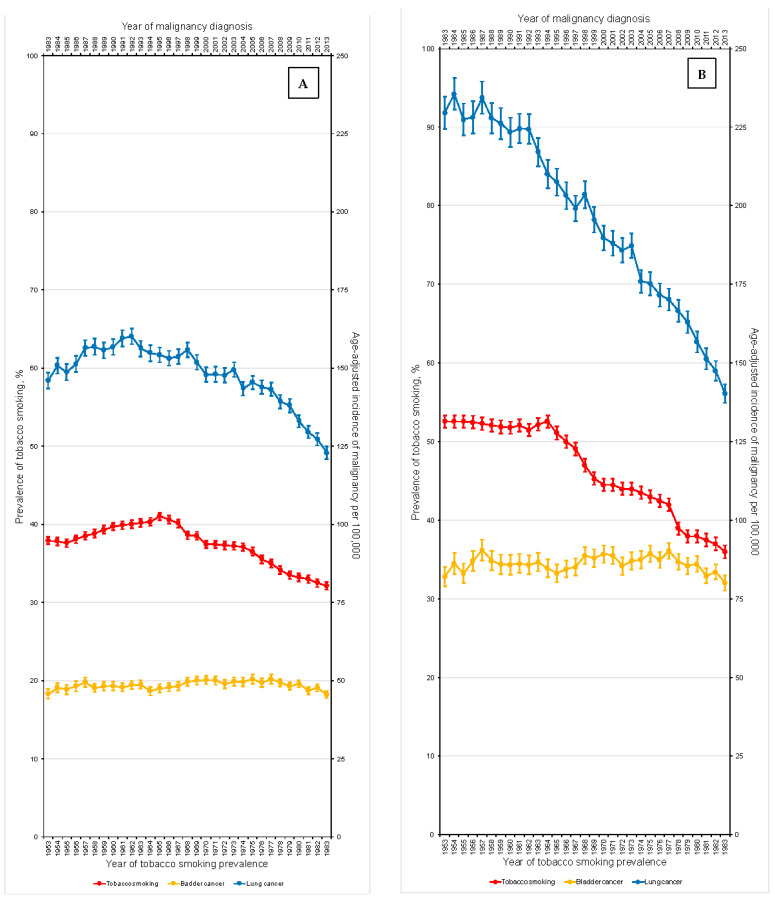

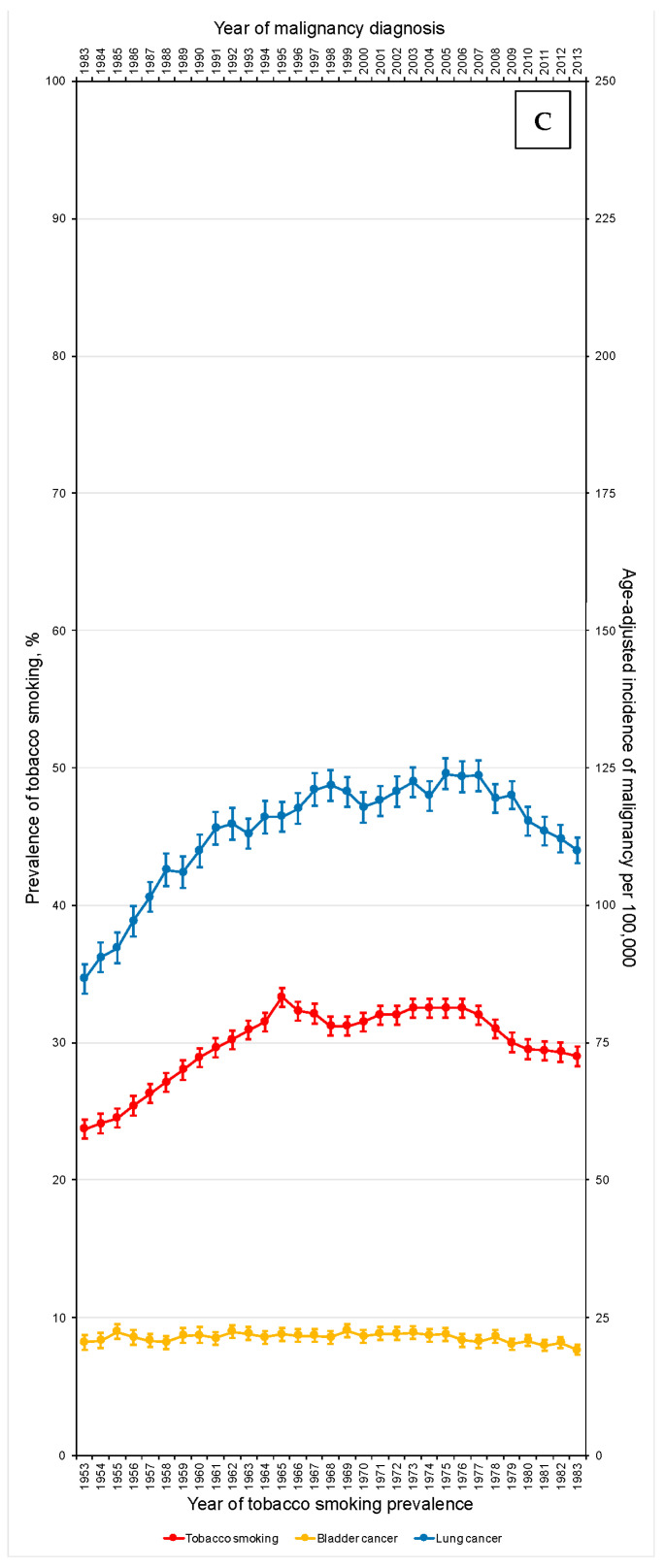
Impact of increasing tobacco smoking prevalence on bladder and lung cancer incidence in the (**A**) overall, (**B**) male, and (**C**) female United States populations over the past-half century.

Between 1953 and 1983, tobacco smoking decreased from 37.9% to 32.1% (APC: −0.22%; 95%CI: −0.29; −0.15; *p* < 0.001). Between 1983 and 2013, the age-adjusted incidence rates for bladder cancer varied non-significantly from 45.6 to 45.5 per 100,000 person-years, respectively (APC: +0.04%; 95%CI: −0.07; +0.16, *p* = 0.43). Over a similar period, the age-adjusted incidence rates for lung cancer decreased from 146.0 to 122.9 per 100,000 person-years, respectively (APC: −0.61%; 95%CI: −0.79; −0.43, *p* < 0.001). All trends are depicted in Figure 1A.

#### 3.1.2. Incidence Rate Differences for Bladder and Lung Cancer

**Figure 2:**
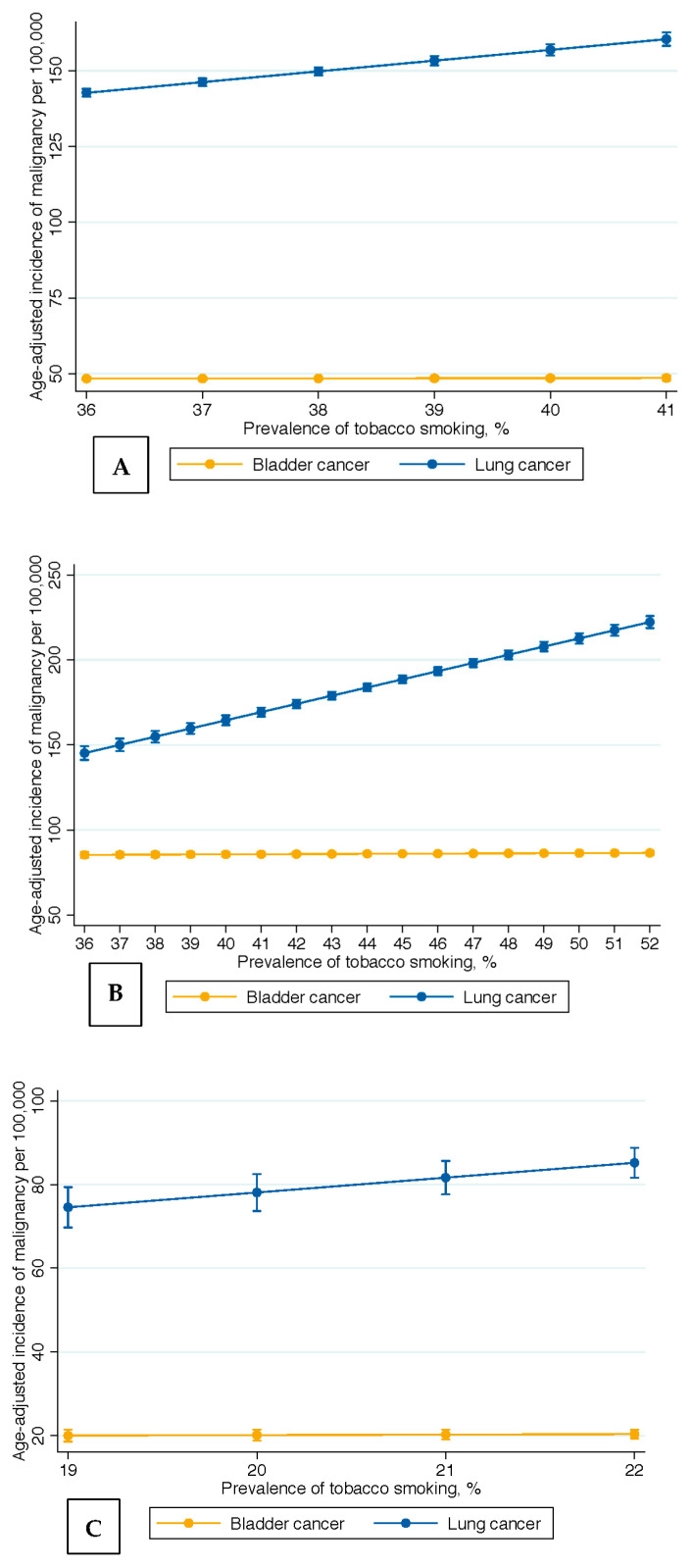
Impact of increasing tobacco smoking prevalence on bladder and lung cancer incidences in the (**A**) overall, (**B**) male, and (**C**) female United States populations over the past-half century.

**Figure 3:**
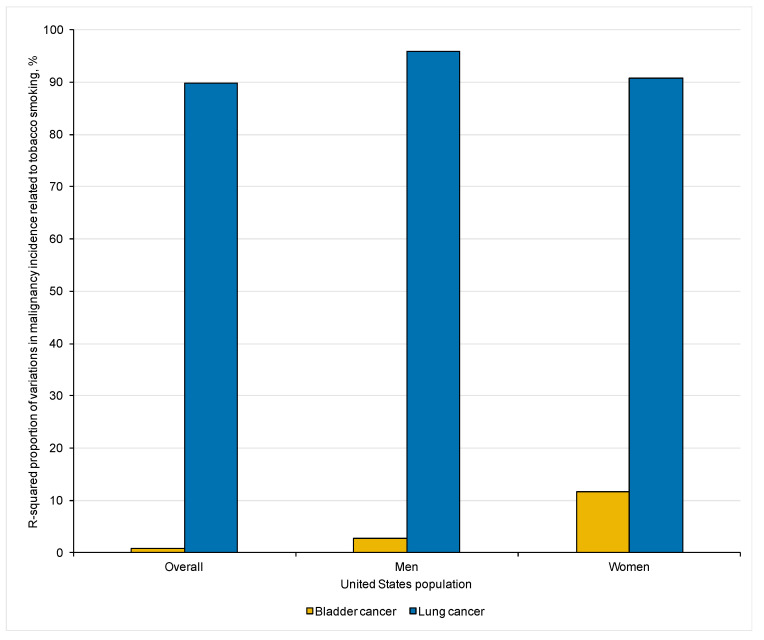
Proportions of variations in bladder and lung cancer incidence related to tobacco smoking exposure in the overall, male, and female United States populations over the past-half century.

The trend in the prevalence of tobacco smoking was significantly associated with the incidence of lung cancer (IRD: +3.55; 95%CI: +3.09; +4.00, *p* < 0.001), but not bladder cancer (IRD: +0.04; 5%CI: −0.14; +0.22, *p* = 0.63; Figure 2A). The comparison between the observed associations in the prevalence of tobacco smoking with the incidence of bladder versus lung cancer was significant (*p* < 0.001). Tobacco exposure was estimated to account for 89.72% and 0.81% of the variation in lung and bladder cancer incidence rates, respectively (Figure 3). The APCs, IRDs, and R squared results in the overall United States population are summarized in Table 1.

### 3.2. Gender-Specific

#### 3.2.1. Annual Percent Changes in Tobacco Smoking Prevalence and Cancer Age-Adjusted Incidence Rates

Between 1953 and 1983, the prevalence of tobacco smoking decreased from 52.6% to 30.0% among men (APC: −0.61%; 95%CI: −0.68; −0.54; *p* < 0.001, Figure 1B) and increased from 23.7% to 29.0% among women (APC: +0.18%, 95%CI: +0.09; +0.27; *p* < 0.001, Figure 1C). 

Between 1983 and 2013, the age-adjusted incidence rates for bladder cancer varied non-significantly from 82.1 to 80.1 per 100,000 person-years among men (APC: −0.04%; 95%CI: −0.17; +0.08; *p* = 0.49, Figure 1B) and slightly decreased from 20.6 to 19.2 per 100,000 person-years among women (APC: −0.18%; 95%CI: −0.33; −0.02; *p* = 0.03, Figure 1C). Over the same period, the age-adjusted incidence rates for lung cancer decreased from 229.6 to 140.3 per 100,000 person-years among men (APC: −1.62%; 95%CI: −1.76; −1.49; *p* < 0.001, Figure 1B) and increased from 86.6 to 109.9 per 100,000 person-years among women (APC: +0.59%; 95%CI: +0.31; +0.88; *p* < 0.001, Figure 1C).

#### 3.2.2. Incidence Rate Differences

Among men, the decreasing trend in the prevalence of tobacco smoking was significantly associated with the trend in the incidence rate of lung cancer (IRD: +4.82; 95%CI: +4.44; +5.20, *p* < 0.001, Figure 2B), but not with the trend in the incidence rate of bladder cancer (IRD: +0.07; 95%CI: −0.09; +0.23; *p* = 0.37, Figure 2B).

Among women, the increasing trend in the prevalence of tobacco smoking was significantly associated with the trend in the incidence rate of lung cancer (IRD: +3.55; 95%CI: +3.12; +3.99; *p* < 0.001, Figure 2C), but not with the trend in the incidence rate of bladder cancer (IRD: +0.12; 95%CI: −0.01; +0.25; *p* = 0.06, Figure 2C).

The comparison between the observed associations in the prevalence of tobacco smoking with the incidence rates of bladder versus lung cancer was significant for both men and women (both *p* < 0.001). Tobacco exposure was estimated to account for 95.80% and 90.69% of the variation in lung cancer incidence in men and women, respectively, while the corresponding proportions for bladder cancer incidence were 2.74% and 11.59%, respectively (Figure 3). APCs, IRDs, and R squared results in the male and female US populations are summarized in Table 1.

## 4. Discussion

The objective of this ecological study was to evaluate the association between trends in the prevalence of tobacco smoking and incidence rates for bladder versus lung cancer. While tobacco smoking is undeniably a risk factor for lung and bladder cancer, our findings suggest that trends in tobacco smoking explain most of the trends in the incidence rates of lung cancer, but not of bladder cancer. In other words, the trends in the incidence rate of lung cancer followed a similar trend to the prevalence of tobacco smoking, while the trend in the incidence rate of bladder cancer followed an independent course from trends in the prevalence of tobacco smoking. Moreover, similar findings were observed for gender-specific data. Therefore, this study provides evidence that tobacco smoking might not explain the totality of the incidence of bladder cancer [23,24]. Based on the literature, other factors that might have influenced trends in the incidence rate of bladder cancer include (1) cigarette composition and smoking behavioral patterns, (2) chemical, environmental, or occupational exposures, and (3) the different pathophysiology and latency of the disease compared with other solid tumors.

First, changes in the composition of cigarettes might explain why, despite a decreasing trend in the prevalence of tobacco smoking, the incidence rate for bladder cancer is stable compared to the decreasing incidence rate in lung cancer. It is postulated that a greater individual risk of developing bladder cancer in today’s smokers may have diluted or even offset the effect of the declining prevalence of tobacco smoking. In 2001, Freedman et al. conducted a large prospective study to measure the risk between tobacco smoking and bladder cancer [25]. Over a 4,518,941 person-years follow up, the authors found that current smokers had a four-fold higher risk of bladder cancer than never smokers—a substantially higher relative risk than the one reported in a seminal meta-analysis (relative risk: 2.94, 95% CI: 2.45–3.54) that examined the effect of smoking on bladder cancer between 1963 and 1987. The authors have attributed this difference to changes in cigarette composition over the past 50 years [23,25]. The concentration of tar and nicotine contained in cigarettes may have decreased, whereas the concentration of carcinogens associated with bladder cancer, such as ß-napthylamine and tobacco-specific nitrosamine, may have increased. These aromatic amine compounds cause DNA damage, including bulky adduct formation in key cancer-related genes, single- or double-strand breaks, and base modifications [26].

In the 1960s, targeted advertisement of cigarettes to women led to a rise in the proportion of female smokers [27]. Whereas, during the same period, the prevalence of tobacco smoking among men decreased. Based on the tobacco composition hypothesis, the incidence of bladder cancer should have increased for women, since the proportion of female smokers has increased and they have most likely been smoking cigarettes with more carcinogens. Instead, the incidence of bladder cancer in women remained generally stable over time—even decreasing slightly toward the end of the study period. Therefore, our gender-specific findings suggest that other factors might have contributed to trends in the incidence rate of bladder cancer [28].

Second, while tobacco smoking and cigarette composition are important risk factors, trends in the incidence of bladder cancer might be influenced by trends in occupational exposures [24]. While evidence for the association between occupational exposure and bladder cancer risk have been documented from as early as the 1940s, awareness and policies to regulate environmental exposure took decades to be implemented [24]. Aromatic amines, including 2-naphthylamine and benzidine, are chemicals that have been associated with occupational exposures among mechanics, painters, and textile workers—occupations that have been historically dominated by a male workforce. Teoh et al. used the World Health Organization Global Health Observatory database to examine gender-specific incidence and mortality trends in bladder cancer. They found that tobacco use was weakly associated with bladder cancer incidence among men (r = 0.20), but strongly associated with bladder cancer incidence among women (r = 0.67) [29]. Thus, occupational exposures—more prominent among men than women—might explain why, despite a decreasing trend in the prevalence of tobacco smoking among men, the incidence rate for bladder cancer is stable.

Moreover, the risk of secondhand smoking was previously dismissed [27]. The harmful effects of secondhand smoking were first described in the 19th Surgeon General’s report on The Health Consequences of Involuntary Smoking in 1986, but it was not until 1993 that we acknowledged that secondhand smoking is responsible for 3000 lung cancer deaths yearly in the United States [27]. While the effect of secondhand smoking on lung cancer has been elucidated, data on its effect on bladder cancer is less well documented. A recent meta-analysis demonstrated that lifetime secondhand smoking exposure is associated with a 22% increased risk for bladder cancer [2].

Third, when accounting for a 30-year latency period, the trends in the prevalence of tobacco smoking mirror trends in the incidence rate of lung cancer but not of bladder cancer. Thus, another explanation of our findings could be that the latency period might be either significantly shorter or significantly longer than the latency observed for lung cancer, preventing us from capturing this trend. To note, studies on the minimum latency of solid tumors are scarce and are often derived from statistical models used to estimate cancer incidence from lifetime exposures to ionizing radiation [30]. Additionally, latency in the development of a malignancy is also related to the genetic predisposition and the extent and type of carcinogen exposure. Thus, a difference in the pathophysiology of lung and bladder cancer might explain the difference in the association between smoking patterns and cancer incidence.

From a practical perspective, the findings of the current study are not meant to suggest a marginal role for smoking in the risk of bladder cancer development. Far from it—there is a significant number of studies in the literature that have examined the direct effects of smoking and the risk of acquiring this lethal disease at the individual level; the consensus has always been clear, and it is that smokers (current or former) are more likely to be diagnosed with bladder cancer than never smokers [6,11,31,32]. Additionally, there is evidence that the relative risk of smoking is higher for lung than for bladder cancer, which might impact the IRD, a measure of the absolute difference in the risk of cancer between smokers and non-smokers [33,34]. This might explain why our observations highlight a lack of congruence between tobacco smoking and the incidence of bladder cancer compared to that of lung cancer. Nevertheless, the takeaway message is that other risk factors may be at play for bladder cancer. Essentially, the identification of these risk factors will be critical in the upcoming years to reduce bladder cancer incidence and mortality rates. Specifically, in addition to some specific individual genetic predispositions—such as the slow *n*-acetyltransferases 2 acetylation phenotype [35]—risk factors of interest certainly include, but may not be limited to, occupational exposure to aromatic amines, iatrogenic factors such as the use of cyclosphosphamide or pelvic radiation therapy [36], water contaminants (i.e., arsenic) [37], or decreased fluid intake [38]. Biologically, the contribution of other risk factors beyond smoking is fully supported by the findings of the differing APOBEC B3 mutation signature (Cytidine deaminase driven signature) that predominates in bladder cancer [39], while lung cancer is more typically characterized by the transversion mutation type [40].

Our results are not devoid of limitations. First, since this is an ecological study, data on individual people are not available (including adjusting for pack-years smoked), and therefore we do not know if the smokers captured between 1953 and 1983 in this study developed bladder or lung cancer. Second, we have not accounted for the pathology of bladder and lung cancer, as tobacco smoking might variably account for the type of pathology. Third, although SEER is a population-based dataset that is representative of the US population, it might oversample for Non-Hispanic American Indian/Alaska Native [41]. Fourth, we relied on historical data abstracted from the Report of the Surgeon General (1950–1978) and the CDC website to obtain historical trends in tobacco smoking prevalence (from 1953 to 1983). This information may be of secondary quality and accuracy given the absence of systematic yearly reports in those years, which were only fully implemented in the 1980s. As such, we mainly estimated the rates of current smokers by using the trend curves available on the CDC website in order to approximate the data, as done previously [40]. Fifth, based on the ‘tobacco epidemic’ theory [22], we chose a 30-year latency period between tobacco smoking and the incidence rates of both malignancies, but we acknowledge that the latency period may be different for bladder and lung cancers. That being said, according to the American Cancer Society, the average age at initial diagnosis of bladder and lung cancers is 70 and 73 years old, respectively. Sixth, we could not adjust for other lifestyle behaviors, environmental factors, occupational risk factors, or tobacco intensity in the incidence trends. Seventh, women are more likely than men to experience a delayed time to clinical investigation following the presence of hematuria, which may lead to suboptimal detection rates of bladder cancer in this population [42,43]. As such, the incidence of bladder cancer in women may have been underestimated, which could have accounted for its slight decrease thirty years after increases in tobacco smoking prevalence. However, the discrepancy in bladder cancer detection between men and women should not have changed over time.

## 5. Conclusions

In contrast to lung cancer incidence, our study showed that variations in tobacco smoking prevalence only partially explained the incidence trends for bladder cancer in the United States population over the past half-century. While smoking is undeniably associated with bladder and lung cancer, our findings suggest that other risk factors may contribute to the incidence of bladder cancer, in addition to tobacco smoking. This highlights the need to further investigate the potential impact of environmental and occupational exposures, as well as genetic predispositions, on bladder cancer risk. Additionally, the study provides evidence that increased awareness of bladder cancer risk factors is necessary in order to reduce bladder cancer incidence and mortality rates.

## Figures and Tables

**Table 1:** Annual percent changes, incidence rate differences, and R-squared statistics in the overall, male, and female United States populations.

Population	Annual Percent Change		Incidence Rate Difference		R-Squared (%)
	%; 95% CI (LB; UB)	*p*-Value	*n*, 95% CI (LB; UB)	*p*-Value	
Overall					
Tobacco smoking prevalence	−0.22; (−0.29; −0.15)	<0.001	-	-	-
Bladder cancer incidence	+0.04; (−0.07; +0.16)	0.43	+0.04, (−0.14; +0.22)	0.631	0.81
Lung cancer incidence	−0.61; (−0.79; −0.43)	<0.001	+3.55, (+3.09 +4.00)	<0.001	89.72
Men					
Tobacco smoking prevalence	−0.61; (−0.68; −0.54)	<0.001	-	-	-
Bladder cancer incidence	−0.04; (−0.17; +0.08)	0.49	+0.07, (−0.09; +0.23)	0.374	2.74
Lung cancer incidence	−1.62; (−1.76; −1.49)	<0.001	+4.82, (+4.44; +5.20)	<0.001	95.80
Women					
Tobacco smoking prevalence	+0.18; (+0.09; +0.27)	<0.001	-	-	-
Bladder cancer incidence	−0.18; (−0.33; −0.002)	0.03	+0.12, (−0.01; +0.25)	0.061	11.59
Lung cancer incidence	+0.59; (+0.31; +0.88)	<0.001	+3.55, (+3.12; +3.99)	<0.001	90.69

## Data Availability

The data is publicly available and was retrieved from the Report of the Surgeon General (Appendix: Cigarette smoking in the United States, 1950–1978; https://profiles.nlm.nih.gov/ps/access/NNBCPH.pdf (accessed on 9 November 2022)) and the Centers for Disease Control (CDC) websites (http://www.cdc.gov/tobacco/data_statistics/tables/trends/cig_smoking/ (accessed on 9 November 2022)) (http://www.cdc.gov/mmwr/preview/mmwrhtml/mm4843a2.htm (accessed on 9 November 2022)).
